# The foundress’s dilemma: group selection for cooperation among queens of the harvester ant, *Pogonomyrmex californicus*

**DOI:** 10.1038/srep29828

**Published:** 2016-07-28

**Authors:** Zachary Shaffer, Takao Sasaki, Brian Haney, Marco Janssen, Stephen C. Pratt, Jennifer H. Fewell

**Affiliations:** 1 School of Life Sciences, Arizona State University, Tempe, AZ 85287, USA; 2 Department of Zoology, University of Oxford, Oxford OX1 3PS, UK; 3 School of Sustainability, Arizona State University, Tempe, AZ 85287, USA

## Abstract

The evolution of cooperation is a fundamental problem in biology, especially for non-relatives, where indirect fitness benefits cannot counter within-group inequalities. Multilevel selection models show how cooperation can evolve if it generates a group-level advantage, even when cooperators are disadvantaged within their group. This allows the possibility of group selection, but few examples have been described in nature. Here we show that group selection can explain the evolution of cooperative nest founding in the harvester ant *Pogonomyrmex californicus*. Through most of this species’ range, colonies are founded by single queens, but in some populations nests are instead founded by cooperative groups of unrelated queens. In mixed groups of cooperative and single-founding queens, we found that aggressive individuals had a survival advantage within their nest, but foundress groups with such non-cooperators died out more often than those with only cooperative members. An agent-based model shows that the between-group advantage of the cooperative phenotype drives it to fixation, despite its within-group disadvantage, but only when population density is high enough to make between-group competition intense. Field data show higher nest density in a population where cooperative founding is common, consistent with greater density driving the evolution of cooperative foundation through group selection.

The relevance of group selection to the evolution of sociality remains contentious^1^,^2^,^3^,^4^,^5^. Eusociality, in which most group members give up direct fitness to contribute to the reproduction of another, seems to have evolved exclusively in family groups^6^. Thus, social evolution in these contexts fits well with inclusive fitness models built around genetic relatedness. In contrast, cooperative social systems, such as communal and joint-nesting societies, are built around a different social context; they are composed of multiple adult individuals performing social behaviors that benefit both themselves and the group as a whole^7^,^8^. In a subset of these societies, group members are non-relatives, and the fitness effects of cooperation are less well accounted for by inclusive fitness models, which focus on the individual level^9^. For these groups, multilevel selection models, like Maynard Smith’s haystack scenario^10^ and David Sloan Wilson’s trait-group models^11^ may provide a more revealing approach, because they better accommodate partitioning of selection between individual and group-level fitness effects^12^,^13^,^14^,^15^.

Both inclusive fitness and multilevel models agree that cooperation could theoretically evolve via group selection when competition between groups is sufficiently stronger than that within groups^16^. Artificial selection experiments have supported this assertion by creating conditions of high between-group competition where individual success has a negative effect on group-level success^17^,^18^,^19^. However, there is a notable scarcity of similar examples in natural populations^15^,^20^,^21^, and controversy about those that have been put forward, as seen in a recent case from social spiders^20^,^22^,^23^,^24^,^25^. This scarcity weakens the argument that group level selection components could be a significant contributor to the evolution of cooperative sociality.

A long-recognized candidate for group selection in nature is cooperative colony foundation in the social hymenoptera, especially ants^9^,^26^,^27^,^28^. In most ants colonies are initiated by a single queen (haplometrosis), but some species show cooperative foundation by groups of queens (pleometrosis) that are typically unrelated^28^,^29^,^30^,^31^. Foundation is a critical life stage, which most colonies do not survive, making it an important context for selection^32^,^33^. Cooperative founding may allow colonies to overcome physiological and ecological constraints that make solitary foundation challenging. However, it also poses costs on individual queens, who must share the colony’s limited reproductive resources with non-relatives. It has been proposed that cooperative founding in some species of ants is driven by intense competition in which nascent colonies raid one another for brood^9^,^26^,^27^,^28^,^30^,^34^. Laboratory evidence shows that pleometrotic groups produce larger workforces earlier, helping them prevail in these contests^30^,^34^. However, a field study found no survival advantage to pleometrotic over haplometrotic groups, and no evidence of brood raiding^35^. Moreover, there is no direct evidence for within-group inequalities generated by cooperation, although division of labor may lead to greater costs for those queens that become foragers^26^,^28^.

To better understand the forces driving cooperation, we studied the harvester ant *Pogonomyrmex californicus*, which is haplometrotic over most of its range, but has some populations where groups of two to fifty or more unrelated queens found nests together^36^. These pleometrotic groups mature into colonies with two or more reproductive queens, marking one of the few cases of primary polygyny in ants^36^,^37^ (Fig. 1b). This polymorphism allows the fitness consequences of these strategies to be experimentally analyzed. Colony foundation can be readily observed in laboratory nests, a technique that has shed light on the development of a colony’s social organization and division of labor^38^,^39^,^40^. This species has also served as a model to investigate both proximate and ultimate questions in the evolution of cooperative behavior^41^. Observation of haplometrotic and pleometrotic colonies points to lower aggression as a key behavioral determinant of the pleometrotic strategy, with haplometrotic queens more likely to escalate conflicts when induced to live in groups^38^,^42^ (Fig. 1a). Aggressive displays in this species, and in ant foundress associations more generally, often escalate quickly into fighting, resulting in the death of one or both queens. Thus, conflict generates a potential differential between individual and group success, such that more aggressive individuals may benefit in contests within groups, but groups with lower aggression have higher survival and subsequent growth.

To measure aggression effects on individual and group-level survival, we collected foundresses during the brief summer period in which new queens emerge from their natal colonies, mate, and found new nests. We sampled two populations, one predominantly haplometrotic and the other predominantly pleometrotic. Previous work has shown that queens from the two populations are similar in size and weight and in their readiness to aggregate and form stable nesting groups^36^,^42^. However, aggression toward co-foundresses is much more likely in queens from the haplometrotic population^36^,^42^. In laboratory observation nests we created six-member foundress associations of two types: 1) pure groups in which all queens came from the pleometrotic population, and 2) mixed groups with one queen from the haplometrotic population. With these combinations we could ask how cooperative individuals fared compared to non-cooperative ones within groups, as well as how fully-cooperative groups fared compared to groups containing non-cooperative individuals. We also made nests with mixed pairs, as well as solitary foundresses of each type to compare the effects of social versus solitary nest founding. We observed the queens for 60 days as they excavated nests and reared their first workers. We made daily records of queen survival, brood presence, and any aggressive behavior.

## Results and Discussion

Survival analysis showed significantly lower individual mortality in groups compared to solitary foundresses, consistent with a survival advantage to cooperation (Supplementary Fig. 1, Supplementary Table 1). In addition, pleometrotic queens in groups of six had higher mortality than their haplometrotic nestmates, as expected if cooperative behavior is at a disadvantage within foundress associations. However, contrary to expectations, pleometrotic queens in mixed groups fared no worse than queens in pure pleometrotic associations, and there was no discernible effect of queen type on survival in foundress pairs (Supplementary Fig. 1, Supplementary Table 1). Behavioral observations revealed a distinct polymorphism between aggressive individuals that attacked their co-foundresses and tolerant individuals that did not. The aggressive type was more common in ants from the haplometrotic region (Table 1), but was also found in ants from the pleometrotic region (Supplementary Table 2, Supplementary Fig. 2). Furthermore, mortality due to aggression (14% overall) was indistinguishable across treatments (χ^2^ = 0.068, df = 3, p = 0.993). Aggression was an important component of queen mortality, with 46 of 229 total deaths (20%) clearly due to aggression (a fact ascertained by the presence of severed heads and abdomens).

**Figure 1:**
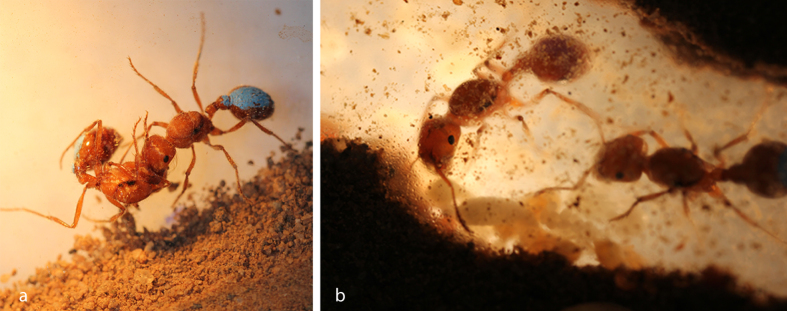
Queens vary in their nest-founding behavioral phenotype. In most of their range, *P. californicus* queens display aggression toward conspecific queens (**a**), leading to solitary nest-founding and mature colonies that are monogynous. But in some populations of this species groups of foundresses cooperate and rear brood together (**b**), leading to mature colonies with multiple queens.

**Figure 2:**
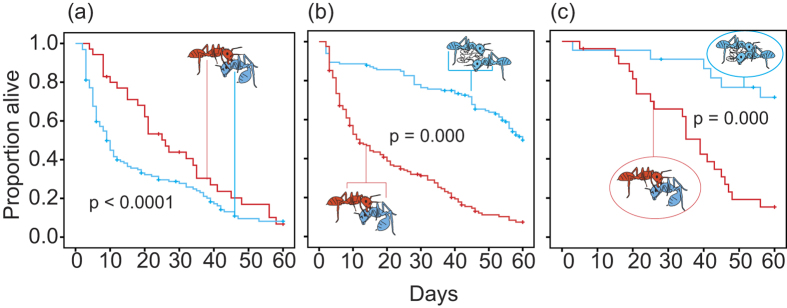
Aggression enhances survival within groups but reduces it between groups. In groups where aggression was documented, aggressive queens (n = 35) survived longer than their non-aggressive co-foundresses (n = 127) (**a**). Individual survival for all queens in nests with aggression (n = 162) was lower than for queens in nests without aggression (n = 132) (**b**). Groups where aggression occurred (n = 27) had lower survival than groups without aggression (n = 22) (**c**). Of the 27 groups that showed evidence of aggression,16 were purely pleometrotic and 11 were mixed. Of the 22 groups that showed no aggression, 14 were pure and 8 were mixed. Crosses on each curve represent censoring times for queens that were not observed for the entire 60-day period.

These results suggest that aggressive and tolerant phenotypes coexist in both populations, although in different proportions (Fig. 1). We therefore reasoned that observed behavioral phenotype (aggressive vs. tolerant) would be a more revealing classification than collection site. When the data were re-analyzed by phenotype instead of population of origin, the results showed that in groups of six containing one or more aggressive queens, initiators of aggression survived significantly longer than their non-aggressive nest-mates (Fig. 2a). In 15 of 27 nests with aggression, the most aggressive queen was the last survivor. Tolerant queens formed the bulk of the victims of aggression, and only 2 of 35 aggressive queens were clearly killed by a co-foundress. However, individuals in groups with only tolerant queens survived significantly longer than individuals in groups with aggression (Fig. 2b). At the group level, associations with no aggressive members survived significantly longer than those having one or more aggressive queens, with group survival defined as having at least one living queen at the end of the experiment (Fig. 2c). Similar trends were found in survival analysis of foundress pairs (Supplementary Fig. 3).

These results conform to the assumptions of a group selection scenario: the tolerant phenotype is locally disadvantaged within mixed groups, but purely tolerant groups have an advantage over those containing aggressors. We used these empirical data to inform an agent-based evolutionary model of the conditions under which the group-level advantage is expected to favor the tolerant phenotype despite its local disadvantage. In a simulated harvester ant population we tracked the success of both phenotypes through several generations characterized by two phases: foundation and growth. In the foundation phase, new foundresses distributed themselves randomly over a landscape and then aggregated with other foundresses within a specified clustering neighborhood. In each of the resulting groups, aggressive ants initiated pairwise fights that ended with the death of one participant. To capture the observed survival advantage of aggressive queens, they enjoyed a higher probability of winning fights with tolerant queens, but equal probability in fights with each other. In game theoretic terms, we conceptualized the within-group interactions more as a prisoner’s dilemma scenario than as a hawk/dove game (where fights would not expected between cooperators and non-cooperators).

The model’s foundation phase ended with nascent colonies consisting either of a single victorious aggressive queen or a variable number of tolerant queens. In the succeeding growth phase, inter-group competition was modeled through pairwise elimination contests between colonies whose territories overlapped. An assumption of our model was that a colony’s potential for success in a contest rose with queen number to a peak at six queens. This reflected the expected pleometrotic advantage in faster rearing of a workforce and thus greater competitive ability^43^,^44^. At higher queen numbers, we assumed that this advantage declined, in accordance with observations on other species with cooperative nest founding^43^,^44^. At the end of the growth phase, surviving colonies produced a new generation of foundresses to start the cycle again, with each colony contributing an equal share, and offspring queens having the same phenotype as their mother queen.

**Figure 3:**
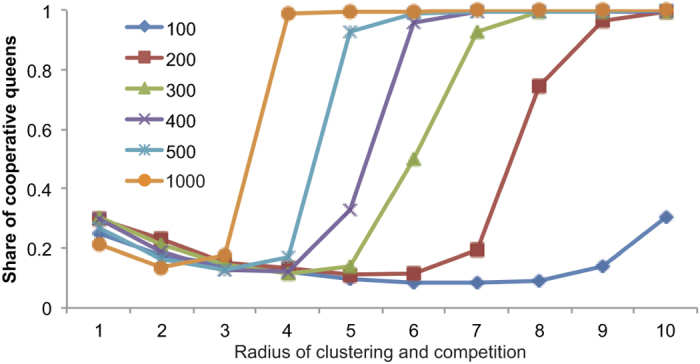
An agent-based evolutionary simulation shows that success of a tolerant foundress phenotype depends on interaction radius and density. An initially rare tolerant phenotype went to fixation only when the distance over which colonies interact surpassed a threshold. This threshold decreased with increasing foundress density, measured as the number of new foundresses produced in each generation. Interaction distance governed both the distance over which foundresses aggregated and the distance over which growing colonies competed. In the simulations shown here, these distances were identical in each simulation, but similar effects were found when they were varied independently (Supplementary Fig. 4).

We tracked the proportion of tolerant individuals over time and found that it increased to fixation only when the area over which each colony interacted with neighbors was sufficiently high. That is, as either the clustering neighborhood or the territory size increased above a threshold value, the outcome switched from favoring the aggressive type to favoring the tolerant type (Fig. 3, Supplementary Fig. 4). Moreover, the location of this threshold depended on population density, with higher densities requiring shorter interaction distances to favor tolerance.

**Figure 4:**
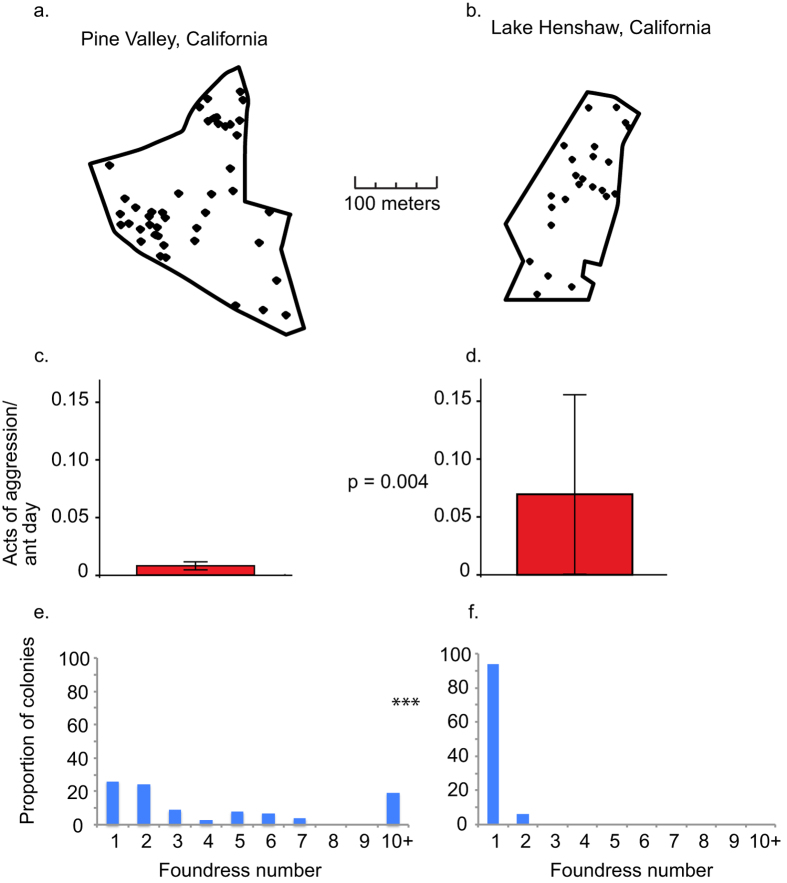
Higher colony density is associated with lower aggression and higher foundress number. Location maps of mature *P. californicus* colonies show higher colony density and clustering in Pine Valley (**a**) than in Lake Henshaw (**b**). Maps were created with the R package *spatstat*^63^. Queens collected from Pine Valley show lower rates of aggression in laboratory foundress associations (**c**) than do queens from Lake Henshaw (**d**). Distributions of natural foundress group sizes show that pleometrosis predominates in Pine Valley (N = 47 nests) (**e**), and haplometrosis in Lake Henshaw (N = 17 nests) (**f**).

The model’s results suggest that population density should be a key determinant of the relative success of the tolerant phenotype, and hence of pleometrotic colony founding. If so, then we would expect to see an association between density and pleometrosis. To test this prediction, we mapped colony locations in sample plots at each of our two collection sites. At Pine Valley where pleometrosis is most common, the sample plot, had 11.9 colonies per hectare, higher than the 9.1 colonies per hectare seen in the Lake Henshaw plot, where nearly all queens found nests solitarily. Previous work has shown that the density of established colonies is associated with the number of new colonies and with foundress number^32^,^33^, suggesting that foundresses experience greater density in Pine Valley. In addition, maps of colony locations (Fig. 4a,b) indicate a more uneven distribution of colonies in Pine Valley, a conclusion supported by quadrat analysis (Supplementary Table 3). This greater clustering implies that colonies in Pine Valley experience more interaction with one another, consistent with the model’s association of inter-colony interaction and pleometrosis.

As noted above, aggressive queens are more common in Lake Henshaw. To examine this more closely, we tabulated acts of aggression in our experimental colonies according to their place of origin and found sharply higher levels of aggression by queens from Lake Henshaw (Fig. 4c,d). To confirm the regional differences in colony founding behavior, we compared our own measurements of foundress group size at Lake Henshaw with similar data collected in Pine Valley by Rissing *et al*.^29^. Pine Valley had significantly more multi-queen nests (35 of 47) than did Lake Henshaw (1 of 17) (χ^2^ = 23.9, df = 1, p = 1.0 × 10^−6^) (Fig. 4e,f). Altogether, these results support a scenario in which greater colony clustering intensifies inter-colony competition, thus favoring the evolution of a tolerant queen phenotype and pleometrotic foundation^32^,^44^,^45^. This conclusion mirrors recent theoretical work that points to the frequency and importance of inter-group interactions as a factor in the strength of group selection^46^,^47^,^48^. Our proposed scenario also resembles the way in which ecological constraints have been proposed to drive the evolution of cooperative breeding in vertebrates^49^. Competitive ability is not the only advantage of pleometrosis^37^,^50^, but the idea that it has a central role in the evolution of foundress associations accords with previous work on other ant species^44^,^45^. Nonetheless, our conclusions about the influence of inter-colony competition on cooperative founding remain tentative, given that we have examined only one population of each type. Stronger inferences must await fuller investigation of multiple haplometrotic and pleometrotic populations.

Our results provide missing empirical support for a group selection model of cooperative behavior in ant foundress associations, validating the intuitions of earlier researchers^9^,^26^,^28^. The value of this finding is not to identify a rare case of group selection acting in nature. Given the fundamental mathematical equivalence of multilevel selection and inclusive fitness models^51^,^52^, all cases of cooperation are potentially explained by partitioning fitness into within- and between-group components. In this view, examples of group selection in nature, far from being scarce, are common-place, even if most scientists have not framed their research this way^53^. Whether to analyze a given problem in an inclusive fitness or multilevel selection framework is largely a question of which is more heuristically useful^51^. The group selection perspective has been productive for many experimental systems and as a tool for agriculture breeders^19^,^54^,^55^,^56^. Furthermore, phenomena ranging from the complex colony-level phenotypes of the social insects to the progression of metastatic cancer are testament to the application of Darwinian principles up and down the hierarchy of life - an idea that can reasonably be attributed to Darwin himself^57^,^58^,^59^,^60^,^61^,^62^.

The scenario we describe for cooperative nest founding in *P. californicus* could be described entirely from an individual perspective, by calculating the expected fitness of each phenotype in each possible combination of group type and environmental setting. However, such an account would obscure the tension between group and individual components of fitness that is especially conspicuous in this system. For one thing, foundress associations constitute a very clear group that acts as a unit of selection above that of the individual queen. This differs from earlier demonstrations of group selection using contextual analysis, where it is often less than clear what the higher unit of selection is^15^,^21^. Furthermore, the fact that queens are unrelated creates a sharper distinction between the goals of individual and group than is seen in another recent report of group selection^24^,^25^. Finally, the simultaneous presence of two distinct behavioral types (cooperative and aggressive) allows us to directly observe a conflict that is largely suppressed in more integrated societies, where the advantages of cooperation and the intensity of group selection weed out such aggressive behavior like a cancer^14^. A leaf-cutter ant society would not last long in competition with other colonies if individual workers often displayed aggressive behavior towards one another or attempted individual reproduction. We can view the behavioral polymorphism of *P. californicus* foundresses as a snapshot in time of what may be a temporary state that soon ends with either stable mutualism or stable aggression. These associations thus allow us to see this fleeting process in a natural context and likely in response to ecological conditions – an on-going tug of war between the selfish objectives of individuals and the competition between groups.

## Materials and Methods

### Queen collection and foundress group observation

Newly mated *Pogonomyrmex californicus* foundresses were collected on July 6–8, 2008 from two neighboring populations in San Diego County, California: 60 were collected at Lake Henshaw (33°14′22″ N, 116°45′46″ W), where haplometrosis predominates, and 324 at Pine Valley (32°46′45″ N, 116°26′56″ W), where pleometrosis predominates. Queens were collected from the ground after they had removed their wings (indicating they were mated) but before they began to excavate nests. They were placed into individual containers and transported to the laboratory, where they were weighed (average mass ± SD = 13 ± 5 mg) and then haphazardly assigned to the following five treatments (sample size refers to number of replicates per treatment):

○ **Single queen**, from haplometrotic population, N = 21.

○ **Single queen**, from pleometrotic population, N = 29.

○ **Mixed pair**, one foundress from each population, N = 20.

○ **Pure group**, 6 foundresses from the pleometrotic population, N = 30.

○ **Mixed group**, 5 foundresses from the pleometrotic population with 1 foundress from the haplometrotic population, N = 19.

Queens were paint marked with a single color on the gaster for individual identification. Each foundress group was introduced to an observation nest comprised of two 15 × 20 cm glass plates separated by plastic sidings slightly wider than the width of an individual queen. Nests were filled with sifted soil from the collection site; they were dampened before use by submersing the bottom in water such that the soil was evenly moist. More water was added whenever the soil appeared dry. Food (approximately 3 grass seeds per foundress) was provided every three days. Nests were housed in a greenhouse with natural day/night cycles. The average temperature was 35 °C during the day and 26 °C at night.

Nests were monitored once a day for two minutes to assess queen survival throughout the 60-day experiment. If aggression was observed, the identities of the participants were noted as well as the intensity and direction of the aggression (for instance, if one ant was biting another). In cases where a queen was found dead with the head or abdomen severed, we considered this to be death from aggression.

### Survival analysis

Effects of queen type and treatment on individual and group mortality were analyzed with Cox proportional hazards models. For analyses that included more than one queen from each group, we modeled group as a random effect, using the R package *coxme*.

### Mapping of colony locations

The locations of all colonies in the two study sites were mapped on June 6–13, 2013. At each site, the same two field technicians walked side by side in a straight line across the southernmost tip of the site, then moved approximately 2 meters north and walked back across the site. This pattern continued until the entire site was surveyed. *P. californicus* colonies were identified by the nest entrance and surrounding cleared area along with noticeable ant activity. The location of each colony was recorded using a Garmin eTrex 10 GPS device. Colonies were only marked when the GPS satellite error was below 3 meters. GPS was also used to ensure that survey paths were walked in straight lines. Surveys were performed when ants are most active, from 8–10 AM or 4–6 PM and at air temperatures between 70–90 °F. When nest entrances were found within 2 meters of each other, an aggression assay was used to determine if they belonged to the same colony.

### Analysis of colony spatial distribution

Location data were analyzed with the R package *spatstat*^63^. Colony density was calculated as the total number of colonies divided by the total area of the surveyed region. For each region, we tested the null hypothesis of random spatial distribution, using the *quadrat.test* function in *spatstat*. This analysis split the study area into quadrats and calculated a χ^2^ statistic from the observed number of colonies in each quadrat vs. that expected if colonies were distributed randomly. Because many of the expected values were less than five, we did not compare the observed statistic with a χ^2^ distribution. Instead we compared it to a distribution generated from 1999 random simulations, using *quadrat.test*’s Monte Carlo method. The test was repeated for a range of quadrat sizes, to avoid being misled by an arbitrary choice of size.

### Measurement of foundress numbers

To quantify foundress numbers at Lake Henshaw, 17 incipient nests were excavated and censused in the field following mating flights on July 1^st^, 2006. These data were compared to similar data collected by Rissing *et al*. at Pine Valley in 2000^29^.

### Agent-based model

The model is implemented in the programmable modeling environment NetLogo^64^. The archived model (code and documentation) is available at the following URL: https://www.openabm.org/model/4262/version/1/view. Agents represent foundresses, and they interact on a torus of N × N cells, with each cell having a probability *p*_*S*_ of being a suitable location for colony foundation.

At the start of each new iteration of the model, there are *m* new queens, giving a density of 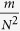 queens per cell. Queens can be aggressive or non-aggressive (i.e. cooperative). The initial share of cooperative queens is *x*_*C0*_.

Queens are distributed randomly on the landscape and then cluster with others nearby. Clustering is implemented by each queen moving to the cell within a radius *r*_*C*_ that has the most other queens on it. The position of the queens is updated in a random order. Clustering behavior is identical for cooperative and aggressive queens, based on the observation that foundresses in the Lake Henshaw and Pine Valley populations show similar readiness to aggregate^42^.

After the clusters are formed, the model evaluates each cell with more than one queen to determine if there are any fights between queens. In random order the model updates each possible pair of queens. An aggressive queen has a probability *p*_*I*_ to initiate fights, and cooperative queens never initiate fights. Each fight ends with the death of one of the two queens. If both queens are aggressive each has a 50% probability to die. If only one queen is aggressive, she dies with probability *p*_*DF*_ (=40%), and her cooperative opponent dies with probability *p*_*DC*_ (=60%). These probabilities are based on mortality outcomes from empirical data^38^. At the conclusion of these within-colony interactions some number of queens remain alive in the group.

When the queens are finished with their internal fights, the effect of inter-group competition is evaluated. Each colony evaluates which other colonies overlap its territory, defined by a radius *r*_*G*_ around each colony. The probability that a colony survives the competition is based on the productivity function of Bartz and Hölldobler^44^:


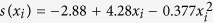


where *x*_*i*_ is the number of queens in colony *i* and *s*(*x*_*i*_) is the number of workers produced. Worker production is assumed to determine a colony’s ability to compete with other colonies, whether by more effective resource use, territorial exclusion, brood raiding^43^,^44^, or other means. Therefore, we use *s*(*x*_*i*_) to define the competitive potential of each colony. In a competition between colonies 1 and 2, the probability that 1 wins the competition, leading to the death of all queens in 2, is equal to *s*(*x*_*1*_)/[*s*(*x*_*1*_) + *s*(*x*_*2*_)]. We assume that colonies that are closer to each other are first to experience the effect of competition. Thus the outcome of competitions is updated in the order of the distance between each pair of colonies with overlapping territories. At the end of the competitions, no territories overlap.

Finally the number of cooperative and aggressive queens in the next generation is calculated. Each colony is allocated the same share of offspring in the next generation, regardless of the number of queens in the colony. For each colony the proportion of cooperative queens is calculated. These values are summed over all colonies, leading to a relative share of cooperation at the landscape level. Then *m* new queens are generated, each one being cooperative with a probability equal to the landscape-level share of cooperation.

After reproduction the mature colonies are removed and the model protocol repeats. Iteration continues until the 200^th^ generation. To ensure that observed outcomes are stable, each new queen can switch to the other type with a probability of *p*_*m*_. Supplementary Table 4 gives the parameter values used in the simulations. The initial fraction of cooperative queens was 0.05. The model was run 100 times for each combination of ten values of the clustering radius *r*_*C*_ and ten values of the competition radius *r*_*G*_. Each combination of *r*_*C*_ and *r*_*G*_ was run at three different population sizes.

## Additional Information

**How to cite this article**: Shaffer, Z. *et al*. The foundress’s dilemma: group selection for cooperation among queens of the harvester ant, *Pogonomyrmex californicus. Sci. Rep.* **6**, 29828; doi: 10.1038/srep29828 (2016).

## Supplementary Material

Supplementary Information

## Figures and Tables

**Table 1:** Frequency of aggression, by population.

Treatment	Aggressive queens	Total queens	Proportion of aggressive queens
P (6P)	23	180	0.13
P (1H:5P)	6	95	0.06
P (1H:1P)	3	19	0.15
H (1H:5P)	6	19	0.32
H (1H:1P)	7	20	0.35
P (all groups)	32	294	0.11
H (all groups)	13	39	0.33

Each row shows queens from one of the two populations (P for pleometrotic and H for haplometrotic), with the treatment group composition in parentheses and the focal type in front of the parenthesis. For example, the row P (1H:1P) tabulates the number of aggressive pleometrotic queens from pairs where the pleometrotic queen was placed with a single haplometrotic partner. Considering all group treatments combined (last two rows), the haplometrotic population contained a higher proportion of aggressive individuals than the pleometrotic population (χ^2^ = 14.8, df = 1, p = 0.0001).
