# Supplementary material for: The foundress’s dilemma: group selection for cooperation among queens of the harvester ant, Pogonomyrmex californicus
Source: Sci Rep. 2016 Jul 28;6:29828. doi: 10.1038/srep29828 (PMC4964563; doi:10.1038/srep29828)
Supplement: Supplementary Information [file srep29828-s1.pdf]

Supplemental material

The foundress's dilemma: group selection for cooperation among queens of the harvester ant, *Pogonomyrmex californicus*

Zachary Shaffer[1*], Takao Sasaki[2], Brian Haney[1], Marco Janssen[3], Stephen C. Pratt[1], Jennifer H. Fewell[1]

[1]School of Life Sciences, Arizona State University, Tempe, AZ 85287, USA

[2]Department of Zoology, University of Oxford, Oxford OX1 3PS, UK

[3]School of Sustainability, Arizona State University, Tempe, AZ 85287, USA

*Correspondence to: Zachary Shaffer, mailing address: 104 E. Garfield St., Tempe, AZ 85281, Email: zshaffe@asu.edu, telephone: 480-294-2901

Figure S1. Outcome of survival analysis for individual queens, classified by experimental treatment and population of origin (pleometrotic (P) or haplometrotic (H)). Queens in groups survived longer than solitary queens. There was no significant difference in survival of single haplometrotic and pleometrotic queens, and little detectable difference in survival among group treatments (see Supplementary Table 1 for details). Curves show Kaplan-Meier estimates of the survival function for each queen type in each treatment. Crosses represent censoring times for queens that were not observed for the entire 60-day period.

Figure S2. Aggressive queens in groups of six ranked by instances of aggression. Aggressive queens were found in both the pleometrotic (black) and haplometrotic (grey) populations.

Figure S3. Survival analysis for mixed pairs of queens (one queen from each population). In pairs where aggression was documented, aggressive queens (red, n=9) survived longer than their non-aggressive co-foundresses (blue, n=9) (a). Individual survival for all queens in nests with aggression (n=18) was lower than for queens in nests without aggression (n=22), although this difference was not statistically significant (b). Groups where aggression occurred (n=9) had lower survival than groups without aggression (n=11), although this difference was not statistically significant (c). Curves show Kaplan-Meier estimates of the survival function for each queen type or pair type. Crosses represent censoring times for queens that were not observed for the entire 60-day period.

Figure S4. Average fraction of cooperative queens over 100 simulations for each combination of competition radius (r_G), cluster radius (r_C), and population size *m* (the number of foundresses in each new generation).

Figure S1

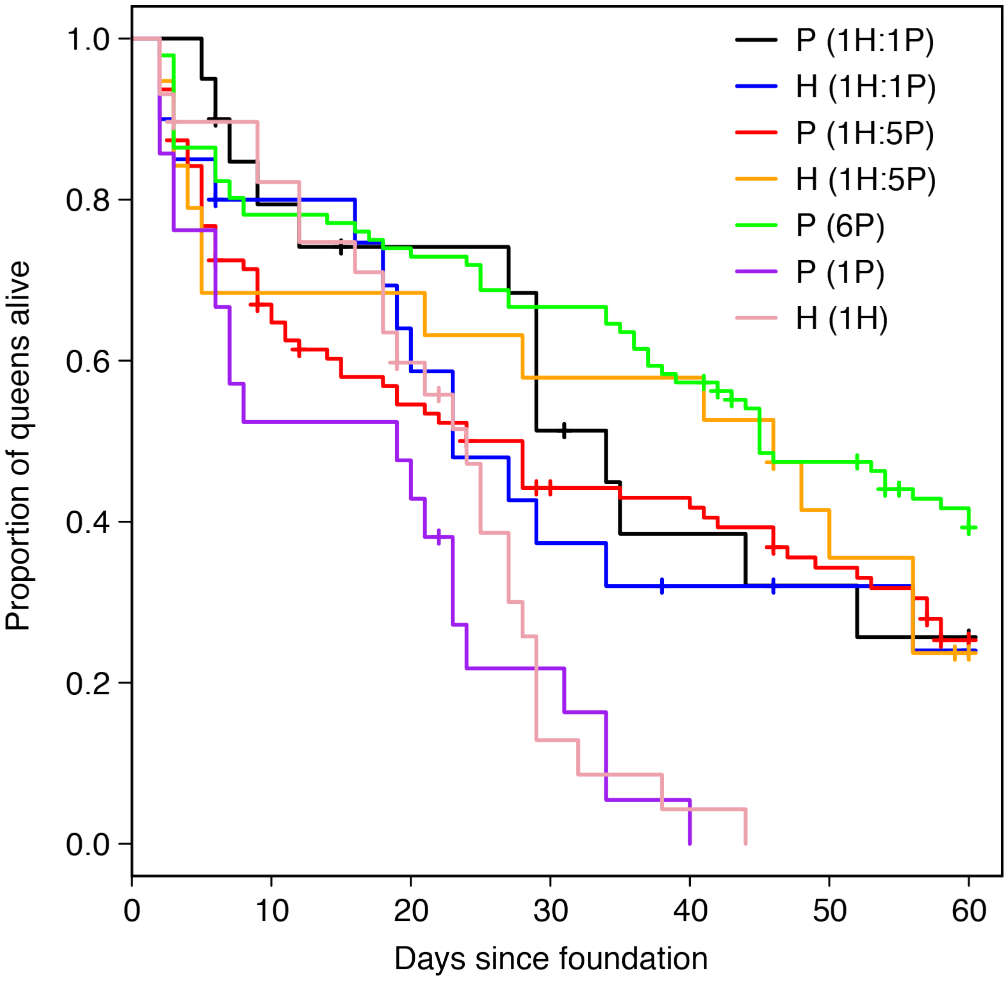
P (1H:1P)
H (1H:1P)
P (1H:5P)
H (1H:5P)
P (6P)
P (1P)
H (1H)
Proportion of queens alive
1.0
0.8
0.6
0.4
0.2
0.0
0
10
20
30
40
50
60
Days since foundation

Figure S2

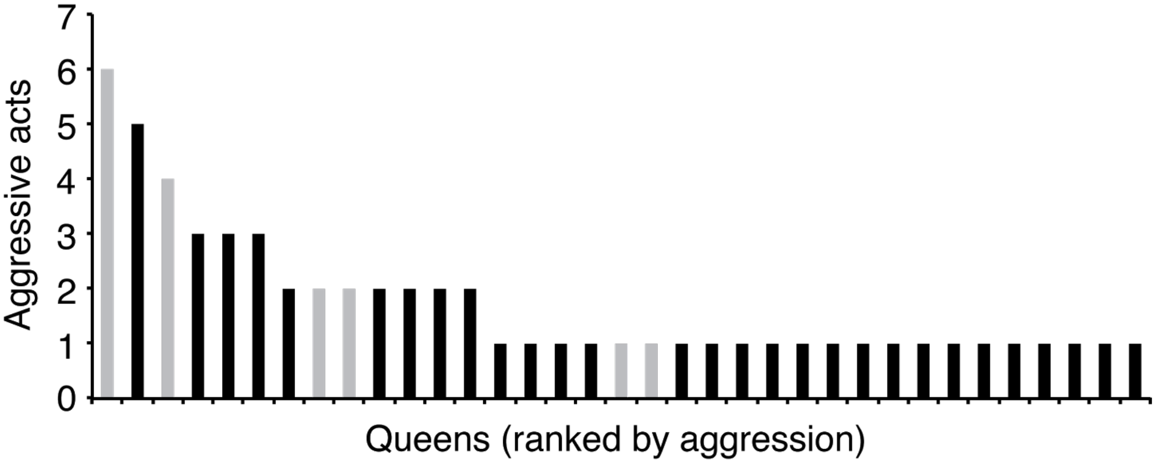

Figure S3

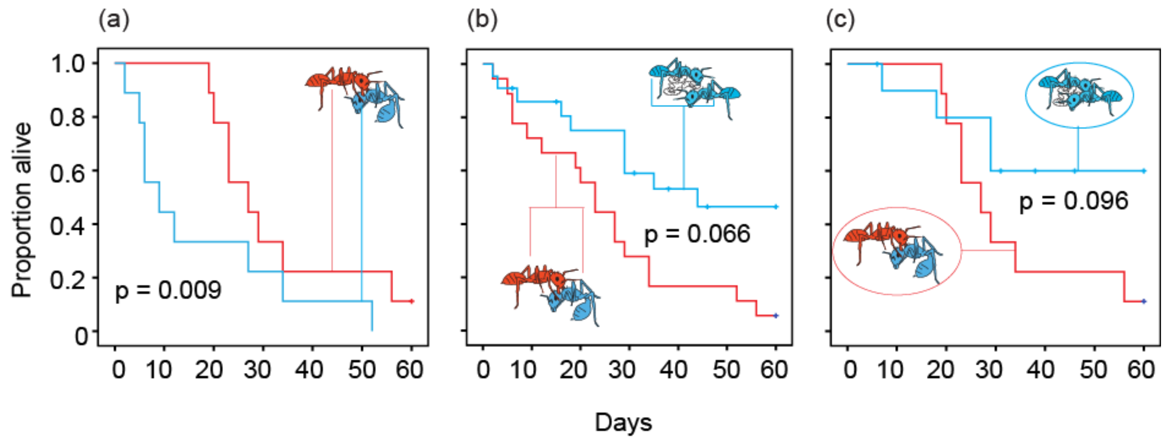
(a)
(b)
(c)
1.0
0.8
0.6
0.4
0.2
0
Proportion alive
p = 0.009
p = 0.066
p = 0.096
0 10 20 30 40 50 60
Days

Figure S4

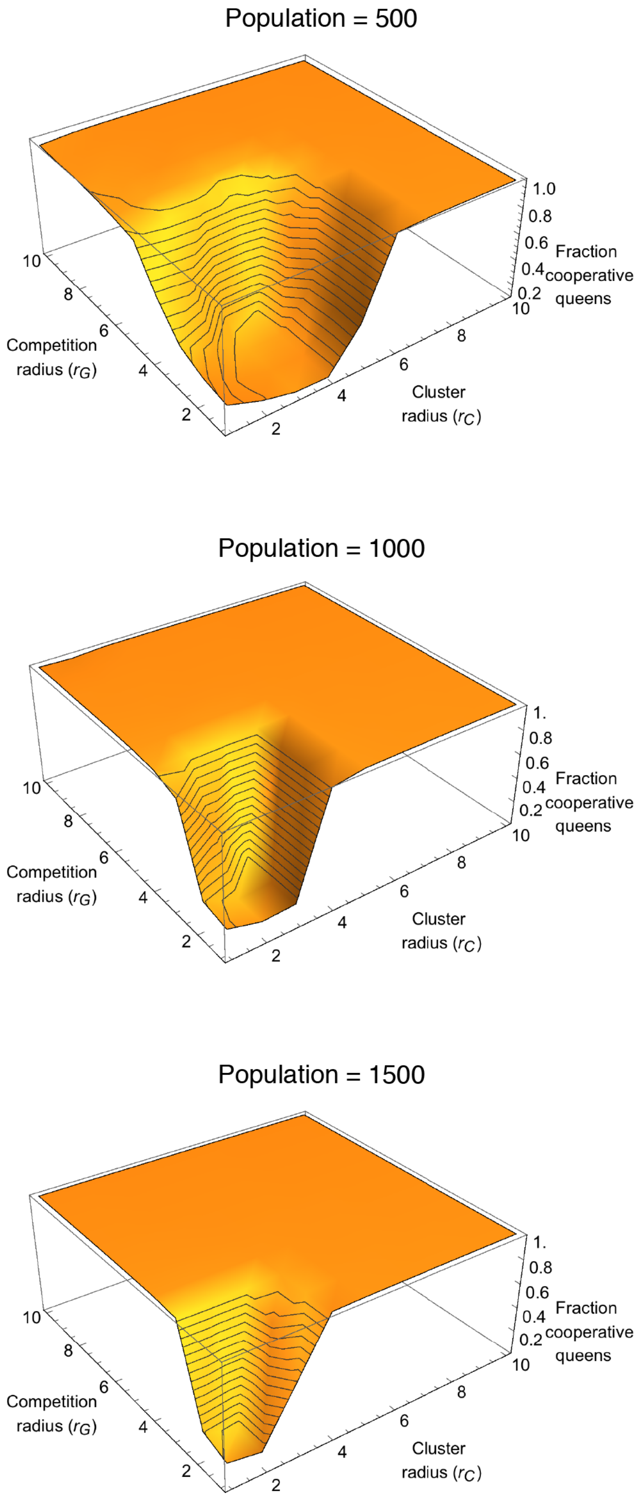

Population = 500
Competition radius (r_G)
Cluster radius (r_C)
Fraction cooperative queens
1.0
0.8
0.6
0.4
0.2
10
8
6
4
2
Population = 1000
Competition radius (r_G)
Cluster radius (r_C)
Fraction cooperative queens
1.
0.8
0.6
0.4
0.2
Population = 1500
Competition radius (r_G)
Cluster radius (r_C)
Fraction cooperative queens
1.
0.8
0.6
0.4
0.2

Supplementary Table 1. Survival analysis of individual queens by population of origin

	N	P (1H:1P)	H (1H:1P)	P (1H:5P)	H (1H:5P)	P (6P)	P (1P)
**P (1H:1P)**	20						
**H (1H:1P)**	20	0.94					
**P (1H:5P)**	95	0.86	0.26				
**H (1H:5P)**	19	0.04	0.45	**2.01**			
**P (6P)**	180	1.2	1.89	1.89	1.27		
**P (1P)**	29	**2.92**	1.83	1.45	**2.98**	**3.72**	
**H (1H)**	21	**3.14**	**2.17**	**2.52**	**3.03**	**4.18**	0.97

Results of Cox proportional hazards models comparing queen survival across treatments. Table entries are the magnitudes of Z scores for pairwise tests of the effect of queen type on survival; bold values indicate significant differences in survival between queen types ($p<0.05$). Row and column labels indicate population of origin (P for pleometrotic and H for haplometrotic), with the treatment group composition in parentheses. For example, P (1H:1P) refers to queens sampled from the pleometrotic population and placed in mixed pairs. The sample size N gives the number of individual queens in each treatment.

Supplementary Table 2. Ant aggression by population and treatment

Treatment	Aggressive acts	Ant-days	Acts / Ant-day
P (6P)	35	5808	0.006
P (1H:5P)	9	2627	0.003
P (all groups of 6)	44	8435	0.005
P (1H:1P)	2	610	0.003
P (all groups)	46	9045	0.005
H (1H:5P)	16	653	0.025
H (1H:1P)	7	548	0.013
H (all groups)	23	1201	0.019

Each row shows aggressive acts per ant-day of observation, for a specific population (H or P) and treatment group (in parentheses). When results are summed for the two populations of origin, queens from the haplometrotic population showed significantly higher aggression rates than queens from the pleometrotic population (χ^2=31.1, df = 1, p = 2.4e-08).

Supplementary Table 3. Spatial analysis of colony distribution in the field

**Lake Henshaw**				**Pine Valley**			
**Number of rows**	**Number of columns**	**P**		**Number of rows**	**Number of columns**	**P**	
2	2	0.03	*	2	2	0.00001	*
2	3	0.44		2	3	0.00972	*
2	4	0.04	*	2	4	0.00001	*
2	5	0.74		2	5	0.02497	*
2	6	0.07		2	6	0.00011	*
3	2	0.13		3	2	0.00019	*
3	3	0.39		3	3	0.14469	
3	4	0.09		3	4	0.00005	*
3	5	0.50		3	5	0.14784	
3	6	0.44		3	6	0.00800	*
4	2	0.23		4	2	0.00017	*
4	3	0.90		4	3	0.12938	
4	4	0.18		4	4	0.00014	*
4	5	0.67		4	5	0.18403	
4	6	0.38		4	6	0.00458	*
5	2	0.56		5	2	0.00003	*
5	3	0.36		5	3	0.33629	
5	4	0.38		5	4	0.00002	*
5	5	0.79		5	5	0.05750	
5	6	0.58		5	6	0.00509	*
6	2	0.43		6	2	0.00011	*
6	3	0.87		6	3	0.30290	
6	4	0.18		6	4	0.00028	*
6	5	0.80		6	5	0.23197	
6	6	0.58		6	6	0.1250	

Each row shows results of a spatial clustering test for a particular number of quadrats, achieved by independently varying the number of rows and columns of quadrats. The pleometrotic population shows statistically significant clustering over a wider range of quadrat sizes than the haplometrotic population.

Supplementary Table 4. Agent-based model parameters

Parameter	Description	Value
N	Dimension of landscape	100
m	Number of new queens	100 - 1500
x_{C0}	Initial percentage of cooperative queens	5
r_C	Radius for clustering of queens	1-10
r_G	Radius for group competition	1-10
p_I	Probability a fierce queen initiate fighting	0 -1
p_s	Probability that a cell is suitable for a colony	1
p_m	Probability of mutation	0.01
p_{DC}	Probability that a cooperative queen dies in a fight	0.6
p_{DF}	Probability that a fierce queen dies in a fight with a cooperative queen	0.4

Values used in simulations are given for each parameter. Where a range of values is indicated, multiple simulations were run at different values in the range.
